# Association of Blood Pressure and Retinal Nerve Fiber Layer Rates of Thinning in Patients with Moderate to Advanced Glaucoma

**DOI:** 10.1016/j.ogla.2024.12.009

**Published:** 2025-01-03

**Authors:** Judy Figueroa, Erica Su, Vahid Mohammadzadeh, Sajad Besharati, Massood Mohammadi, Maryam Ashrafkhorasani, Simon K. Law, Anne L. Coleman, Joseph Caprioli, Robert E. Weiss, Kouros Nouri-Mahdavi

**Affiliations:** 1 Glaucoma Division, Stein Eye Institute, David Geffen School of Medicine, University of California Los Angeles, Los Angeles, California; 2 Department of Ophthalmology, University of Louisville, Louisville, Kentucky; 3 Department of Ophthalmology, Indiana University School of Medicine, Indianapolis, Indiana; 4 Department of Epidemiology, Fielding School of Public Health, University of California Los Angeles, Los Angeles, California; 5 Department of Biostatistics, Fielding School of Public Health, University of California Los Angeles, Los Angeles, California

**Keywords:** Glaucoma, Retinal nerve fiber layer, Intraocular pressure, Prognostic model, Blood pressure

## Abstract

**Purpose:**

To investigate the influence of baseline blood pressure (BP) on retinal nerve fiber layer (RNFL) rates of change (RoCs) in glaucoma patients with central damage or moderate to severe disease.

**Design:**

Prospective cohort study.

**Participants:**

One hundred ten eyes with ≥ 4 RNFL OCT scans and ≥ 2 years of follow-up.

**Methods:**

Global RNFL RoCs were modeled with a Bayesian hierarchical model with subject- and sector-level random effects. Influence of baseline systolic and diastolic BP measures and their interactions with intraocular pressure (IOP) on global RNFL RoCs was investigated in prognostic models adjusting for relevant baseline demographic and clinical measures.

**Main Outcome Measures:**

Magnitude and direction of coefficients for BP, IOP, and their interaction for prediction of global RNFL RoCs. One-sided Bayesian *P* values denote posterior probability that a regression coefficient is greater than or less than zero, with *P* < 0.025 or *P* > 0.975 defining significance.

**Results:**

Average (standard deviation) 24-2 visual field mean deviation (MD) at baseline, follow-up time, and number of OCT scans were −8.8 (6.0) dB, 4.3 (0.5) years, and 8.3 (1.4), respectively. In multivariable analyses, female sex, Hispanic ethnicity (vs. White ethnicity), better baseline 24-2 MD, higher contrast sensitivity at 12 cycles per degree, presence of diabetes, and thicker central cornea predicted faster RNFL thinning. Adjusted for covariates, lower diastolic BP combined with higher IOP predicted faster RNFL RoCs. Parallel multivariable models incorporating systolic BP showed similar effects. Among various BP/IOP combinations, eyes with IOP at the 90th percentile and diastolic (systolic) BP at 10th percentile demonstrated the fastest RNFL thinning rates (−0.554 and −0.539 μm/year).

**Conclusions:**

Low BP and higher IOP at baseline predicted faster (worse) RNFL RoCs in glaucoma patients with central damage or moderate to advanced disease. Although there may be potential benefits to BP management in glaucoma patients, the therapeutic value of BP manipulation in glaucoma patients is yet to be established given the proven benefits of tight BP control in reducing cardiovascular morbidity and mortality.

**Financial Disclosure(s):**

Proprietary or commercial disclosure may be found in the Footnotes and Disclosures at the end of this article.

Glaucoma is a progressive optic neuropathy characterized by changes in the optic nerve configuration and loss of retinal nerve fibers. Intraocular pressure (IOP) is well established as a modifiable risk factor for glaucoma development and progression.^1–4^ Detection of glaucoma progression is essential to properly manage the disease and mitigate vision loss or blindness.^5^ Previous studies explored the influence of baseline blood pressure (BP) on future visual field progression.^4,6–17^ A few prior studies have examined the influence of baseline systemic blood pressure (BP) on structural glaucoma progression.^2,16,18,19^

In a prospective study of 275 patients, Hayreh et al^8^ demonstrated that systemic hypotension and nocturnal BP dips significantly increased the risk of glaucoma progression and proposed that this occurred through ischemia affecting the optic nerve.^8,20^ Similarly, Charlson et al^21^ found that low nocturnal BP may be associated with a higher risk of visual field loss. The vascular theory of open-angle glaucoma (OAG) suggests that blood flow to the optic nerve head decreases when systemic blood pressure is low, especially when IOP is high.^17^ This reduced blood flow or perfusion pressure leads to damage to the retinal ganglion cells. Prior epidemiological studies such as the Barbados Eye Study,^22^ the Rotterdam Study,^23^ the Baltimore Eye Survey,^9^ and the Egna-Neumarkt Study^24^ provided evidence for the association of systemic blood pressure variables with increased incidence of glaucoma. Prospective clinical trials and observational studies found that lower BP is a risk factor for glaucoma progression.^4,16^

In this article, we build upon our prior work describing the predictors for macular ganglion cell complex rates of change (ROCs).^18^ The current study investigates the association between baseline diastolic BP (DBP) and systolic BP (SBP) measurements and retinal nerve fiber layer (RNFL) rates of thinning in glaucoma patients with central or moderate to advanced damage. Our newly developed Bayesian longitudinal model leverages the available data from 12 RNFL sectors to estimate global RNFL ROCs and hence is more powerful in detecting predictors of RNFL RoCs. Although one would expect many predictors to be similar among functional and structural prognostic models, given the fact that structural outcomes are easier to model, we hypothesized that the Bayesian structural prognostic model would be more likely to show previously unidentified predictors or confirm significant known predictors compared with a functional model. We implemented our recently developed Bayesian hierarchical model to construct prognostic models predicting global RNFL RoCs adjusting for baseline covariates.^25^

## Methods

This prospective cohort study enrolled 110 eyes (110 patients) from the Advanced Glaucoma Progression Study. The study was approved by the Human Research Protection Program at the University of California, Los Angeles and was conducted in compliance with the Declaration of Helsinki principles and Health Insurance Portability and Accountability Act policies. Informed consent was obtained from all patients at the time of study enrollment. Data collection for the cohort started in 2012, and analyses are based on the data aggregated in 2020. This study was designed in May 2022 and was finalized in April 2023. The findings were reported in accordance with the Strengthening of the Reporting of Observational Studies in Epidemiology statement checklist.

Inclusion criteria for eyes in the study were (1) diagnosis of primary OAG, pseudoexfoliative glaucoma, pigmentary glaucoma, or primary angle-closure glaucoma and (2) evidence of central damage on 24-2 visual fields (presence of 2 or more points with *P* < 0.05 within the central 10° on 24-2 visual fields^26^) or visual field mean deviation (MD) less than −6 dB. Exclusion criteria included (1) baseline age < 39 years or > 80 years; (2) best-corrected visual acuity < 20/50; (3) refractive error > 8 diopters of sphere or > 3 diopters of cylinder; and (4) any significant confounding ocular, retinal, or neurological disease. All eyes underwent OCT imaging at baseline and every 6 months. Eligible eyes were required to have ≥4 OCT scans and ≥ 2 years of follow-up. If both eyes met inclusion criteria, the eye with worse MD was selected.

All patients had a baseline BP as part of the study protocol. Blood pressure was not used as an inclusion or exclusion criterion. Blood pressure measurements were obtained by a trained research coordinator. An Omron BP monitor (model BP760N) was used for measurements. Participants were seated quietly for 5 minutes before BP was taken. The right arm was preferred for consistency and comparison with the standard tables.

### OCT Imaging

Retinal nerve fiber layer thickness measurements were obtained with a 12° circular peripapillary OCT scan (3.4 mm in diameter in an emmetropic eye) with Spectralis spectral domain OCT (Heidelberg Engineering). Only good quality scans, defined as those with quality scores ≥ 15 and no artifacts, were analyzed. Retinal nerve fiber layer segmentation was checked and manually corrected by 2 independent reviewers (M.M. and V.M.). The data were exported as an XML file providing 768 A-scan measurements; we calculated the clock-hour sectoral thickness measurements by averaging the raw data.

### Statistical Analyses

We modeled RNFL RoCs for 12 clock-hour sectors and globally with a previously described Bayesian hierarchical model, incorporating random effects at both subject and sector levels and relevant covariates.^25^ Our methods for data exploration and cleaning have been previously described.^26^ We identified and removed approximately 1.1% of the observations as outliers. Details of the outlier removal algorithm are provided in the Supplementary Material (available at www.ophthalmologyglaucoma.org). We evaluated profile plots and empirical summary plots and reviewed univariate distributions of covariates.

Our Bayesian model includes 7 interpretable parameters at the sector level: population intercept, population slope, variance of the random intercepts, variance of the random slopes, correlation between random intercepts and slopes, and the mean and standard deviation (SD) of the residual variances. We transformed some of these 7 parameters for ease of prior specification. We assigned each of the sector-level parameters with unknown global mean and SDs. We provide complete details in the Supplementary Material.

We standardized all covariates, including numerical, binary, and categorical ones, by subtracting measurements from the sample mean and dividing by the sample SD of the covariate. For binary variables such as sex, presence of diabetes or hypertension, and treatment of hypertension, an indicator variable was used. For race, we used 3 indicator variables (for Black, Hispanic, and Asian with White as the reference group). Numerical variables consisted of age, central corneal thickness (CCT), IOP, axial length, contrast sensitivity, and 24-2 visual field MD. Standardization of covariates simplified prior specification for coefficients; we back-transformed coefficients before reporting results. Thus, reported results are coefficients of unstandardized covariates. Baseline IOP represented medicated IOP at the time of enrollment in the study. We used a shrinkage prior for all covariate coefficients.^27,28^ We fit models with a single covariate, with a main effect to assess the impact on baseline RNFL and a covariate-by-time interaction to assess the impact on RNFL RoCs. We then fit a multivariable model that included all main covariate effects mentioned above and covariate-by-time interactions. We fit a final model that included IOP and DBP interactions, with the interaction having an association with the baseline value and with the rate of change. Systolic BP was similarly examined using the same model, replacing DBP with SBP. In this final model, for interpreting effects of BP and IOP, the coefficients for other numerical variables were fixed at posterior means and those of the categorical variables at the reference group. Visualization of RNFL change showed no floor effect.

We present posterior means, SDs, and 95% credible intervals for the unstandardized covariate coefficients. Statistical significance was determined using 1-sided Bayesian *P* values, the posterior probability that the regression coefficient was greater (or smaller) than zero. When the Bayesian *P* value was less than 0.025 or greater than 0.975, we declared the coefficient significant at *P* < 0.025. A significant *P* value means we are quite certain a posteriori that we know the sign of the coefficient. All models were fit with JAGS^29^ using the *R2jags* package in R.^30,31^

## Results

One hundred ten patients were included in this study. Among the 110 study participants, 4 (3.6%) eyes were newly diagnosed. Table 1 provides baseline demographic and clinical characteristics of the cohort. The mean (SD) age at baseline was 66.7 (8.6) years, with 62% female participants. The ethnic composition of the cohort was as follows: 57 (51.8%) White 22 (20.0%) Asian, 18 (16.4%) Black, and 13 (11.8%) patients of Hispanic descent. The average (SD) follow-up duration and number of OCT scans were 4.3 (0.5) years and 8.3 (1.4), respectively. The mean (SD) 24-2 visual field MD and IOP at baseline were −8.8 (6.0) dB and 12.6 (3.9) mmHg, respectively. The average (SD) baseline SBP and DBP measurements were 135.1 (18.1) mmHg and 81.9 (10.3) mmHg, respectively. At baseline, 58 (52.7%) eyes were being treated with β-blockers; this number decreased to 19 (17.3%) at the final visit. In addition, the average (SD) number of medications used was 1.6 (1.1) at baseline; this number decreased to 0.6 (1.2) at the final visit. During the follow-up period, 31 (28.2%) eyes underwent trabeculectomy, 7 (6.4%) eyes required trabeculectomy revision, 4 (3.6%) eyes had cataract surgery with iStent implantation, 2 eyes (1.8%) underwent a bleb revision, and 7 (6.4%) eyes received selective laser trabeculoplasty. Before building prognostic models, we carried out correlation analyses between pooled RNFL slopes at 12 sectors and baseline diastolic and systolic BP, IOP, and age (Figures S1–S4, available at www.ophthalmologyglaucoma.org).

Table S1 (available at www.ophthalmologyglaucoma.org) presents the univariate model showing the association of each covariate with estimated RNFL thickness at baseline (RNFL intercepts). Female sex, Asian (vs. White ethnicity), and worse baseline 24-2 MD predicted lower RNFL thickness. Table S2 (available at www.ophthalmologyglaucoma.org) provides summaries from the univariate models for the association of each covariate with RNFL RoCs. Female sex, Hispanic or Asian descent (vs. White), presence of diabetes, thicker CCT, shorter axial length, higher contrast sensitivity at 12 cycles per degree, higher (better) baseline 24-2 MD, and higher IOP predicted faster RNFL thinning. There was no significant correlation between IOP and systemic BP at baseline (*r* = −0.048; *P* = 0.621 for DBP and 0.049; *P* = 0.614 for SBP). Tables S3 and S4 (available at www.ophthalmologyglaucoma.org) demonstrate the influence of the covariates on the RNFL intercepts (estimated baseline RNFL thickness) from multivariable models including the DBP and SBP, respectively.

Table 2 presents the multivariable prognostic model incorporating DBP and including the DBP × IOP interaction on RNFL ROCs. Female sex, Hispanic descent (vs. White ethnicity), presence of diabetes, thicker CCT, higher contrast sensitivity at 12 cycles per degree, and higher (better) 24-2 MD at baseline predicted faster RNFL thinning. The interaction between IOP and DBP was significant; study eyes with lower DBP and higher IOP had steeper RNFL slopes. Table 3 presents the multivariable model incorporating SBP and the SBP and IOP interaction; the significant predictors were the same as the parallel model for DBP. Again, study eyes with lower SBP and higher IOP had steeper RNFL slopes.

**Figure 1.**
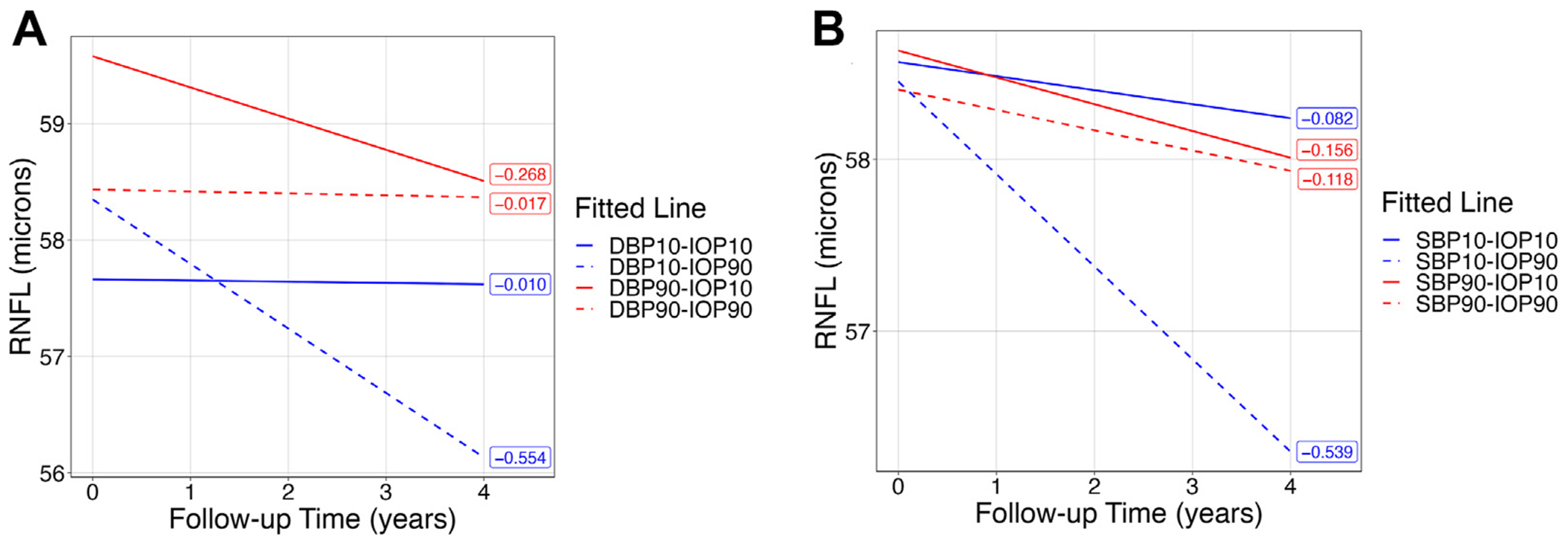
**A,** Fitted regression lines for hypothetical subjects at the 10th and 90th quantiles cutoff for DBP and IOP. **B,** Fitted regression lines for hypothetical subjects at the 10th and 90th quantiles cutoff for SBP and IOP. DBP = diastolic blood pressure; IOP = intraocular pressure; RNFL = retinal nerve fiber layer; SBP = systolic blood pressure.

Figure 1A, B shows the fitted regression lines (RNFL slopes) for hypothetical subjects with all 4 combinations of 10th and 90th sample percentiles for DBP (SBP) and IOP as derived from the multivariable models. A combination of low DBP (SBP) and high IOP at baseline resulted in the most rapid rates of RNFL thinning during the study period. For example, eyes with IOP at the 90th percentile level and DBP (SBP) at 10th percentile level would demonstrate the fastest RNFL thinning rates. Tables S5 and S6 (available at www.ophthalmologyglaucoma.org) present summaries of the RNFL posterior slopes for hypothetical study participants at 4 combinations of 10th or 90th percentiles of DBP (SBP) and IOP. Study eyes with lower DBP and higher IOP had steeper RNFL slopes ranging from −0.554 mm/year (90th IOP and 10th DBP) to −0.010 μm/year (10th IOP and 10th DBP). At IOP cutoff points of 8 (10th percentile) and 16 mmHg (90th percentile), every 10 mmHg lower DBP was associated with 0.106 μm/year and −0.240 μm/year faster RoCs of RNFL, respectively. Tables S7 and S8 (available at www.ophthalmologyglaucoma.org) show the differences in the posterior slopes for the 4 hypothetical combinations of IOP and DBP (SBP). The posterior slopes for the combination of 90th percentile IOP and 10th percentile DBP (SBP) were significantly faster than all the other possible combinations.

## Discussion

We found that baseline BP measures influenced subsequent RNFL RoCs in this cohort of glaucoma patients with central damage or moderate to advanced disease. Specifically, lower baseline DBP *or* SBP measurements were associated with faster rates of RNFL thinning in the presence of higher baseline IOPs. Eyes with the highest IOP and lowest BP at baseline exhibited the fastest rates of RNFL loss during the follow-up. In models incorporating either SBP or DBP, female sex, Hispanic descent (compared with White ethnicity), higher baseline 24-2 MD, higher contrast sensitivity at 12 cycles per degree, presence of diabetes, and thicker CCT were also associated with faster RNFL thinning.

Several studies have addressed the relationship between ocular perfusion pressure (OPP) and glaucoma.^2,9,32,33^ Ocular perfusion pressure, defined as the difference between the mean arterial pressure and IOP, is important in maintaining adequate blood flow to the optic nerve.^18,32,34^ Inadequate perfusion due to vascular dysregulation can compromise blood flow to the eye.^32^ The limiting factor for using OPP as a predictor is that it represents a fixed one-to-one trade-off between BP and IOP; including OPP as a predictor of RNFL RoCs would preclude exploration of interactions between IOP and BP.

The association of systemic BP and IOP with the presence of glaucoma has been investigated in population-based studies including the Barbados Eye Study, Rotterdam Study, Baltimore Eye Survey, Egna-Neumarkt Study, and Thessaloniki Eye study.^9,22–24,35^ The Barbados Eye Study found an association between lower SBP and higher IOP measurements.^22^ The Rotterdam Study reported a relationship between OAG and both higher SBP and DBP.^23^ In the Baltimore Eye Survey, there was an association between low DBP and high IOP and OAG.^9^ The Egna-Neumarkt Study found that lower DBP and higher IOP predicted the presence of glaucoma.^24^ The Los Angeles Latino Eye Study reported that higher SBP and lower DBP were risk factors for the presence of glaucoma.^17^ On the other hand, the Thessaloniki Eye study reported that high SBP or diastolic DBP was a risk factor for primary OAG.^35^

Numerous studies have explored the association between BP and visual field progression in glaucoma patients.^4,6–17^ Visual field progression has been linked to both DBP and SBP; specifically, lower DBP levels are thought to be associated with higher risk of visual field worsening in glaucoma by reducing OPP damaging the optic nerve. However, the role of SBP is more controversial, with conflicting reports in the literature; studies have suggested that either lower or higher SBP levels could negatively influence the course of glaucoma.^4,12,17^ Studies in eyes with primary OAG and normal tension glaucoma found an increased risk of visual field deterioration in patients with nocturnal BP fluctuation > 20% compared with those with fluctuations within the normal range (10%–20%).^6–8^ Systemic low BP and significant nighttime BP dips can lead to a higher risk of glaucoma progression,^8,10^ suggesting ischemic damage to the optic nerve. The Early Manifest Glaucoma Trial reported lower SBP as a contributing factor for glaucoma progression especially in patients with a cardiovascular history and higher baseline IOP.^4^ Chen et al^16^ found that, in a cohort of Chinese patients with untreated normal tension glaucoma, visual field progression was associated with higher baseline IOP and lower minimum DBP.^16^

A few prior studies explored the association between BP and structural progression in glaucoma.^2,16,18,19^ A study by Lee et al^19^ found that in normal tension glaucoma patients, minimum DBP predicted subsequent thinning of the macular ganglion cell-inner plexiform layer, while minimum SBP was more significantly associated with peripapillary RNFL thinning in a decision tree analysis. Jammal et al^2^ reported that lower mean arterial BP and DBP measurements were significantly associated with faster rates of RNFL loss. Chen et al^16^ found that lower minimum DBP was associated with progressive thinning of RNFL and macular ganglion cell-inner plexiform layer in Chinese patients with normal tension glaucoma. Mohammadzadeh et al^18^ reported that subjects with higher IOP and lower DBP had faster rates of ganglion cell complex thinning.^18^ Meanwhile, baseline SBP was only weakly related to ganglion cell complex RoCs in this cohort of patients with central damage or moderate to advanced disease. In the current study, we found that baseline SBP was more prominently related to RNFL RoCs. We speculate that this may be due to the fact that RNFL measurements represent the entire complement of the retinal ganglion cell axons in an individual eye, and hence, the association between the RNFL RoCs and various covariates might be easier to find. The underlying mechanism of the potential influence of BP on glaucoma deterioration is likely related to its effect on tissue perfusion, which is essential for maintaining the health of the optic nerve axons.

Jammal et al^36^ recently demonstrated that higher IOP led to faster rates of RNFL thinning. For each 1 mmHg increase in IOP, RNFL thickness decreased by 0.05 μm/year. Conversely, several mechanisms involving vascular regulatory dysfunction have been proposed to explain how BP influences glaucoma damage. One such mechanism involves reduced ocular blood flow leading to optic nerve ischemia, which is associated with increased levels of endothelin 1, a vasoconstrictor, in patients with glaucoma, further contributing to RNFL thinning.^34,37^ Jin et al^38^ suggested that significant fluctuations in BP exceed the capability of vascular autoregulatory mechanisms, thereby disrupting microcirculation and resulting in damage to the optic nerve. Ocular vascular autoregulation is the process where blood vessels in the eye actively adjust in response to local signals to maintain blood flow and a decrease in BP could disrupt this process.^39^ Therefore, both IOP and BP must be carefully evaluated in glaucoma patients to prevent RNFL thinning and further structural progression. Rajasundaram et al^40^ recently suggested, based on a Mendelian randomization study, that the effect of BP on retinal ganglion cells may be exerted independent of the IOP.

There are limitations to consider regarding the findings of this study. Our sample size was relatively small with only 110 eyes meeting the strict inclusion criteria. A larger sample size may find other associations that might have been missed. We investigated only the influence of baseline covariates including BP and IOP on RoCs. While BP measurements were repeated every year during the study, developing a longitudinal hierarchical Bayesian model to incorporate time-varying variables such as IOP or BP is a major undertaking and will be the topic of a subsequent study. Future research, particularly longitudinal studies with 24-hour BP or IOP readings to monitor the fluctuations in BP and IOP outside the clinical setting, will provide a more comprehensive understanding of their mutual impact on glaucoma progression. Within the range of BP measured in our cohort, we did not see a particularly nonlinear relationship between BP and RNFL change over time based on scatterplots. It is possible that very high BP may be damaging to the optic nerve, but we did not have enough data to test this. Identifying any weak nonlinear relationships would require a rather wider range of BP measurements and a much larger sample size. Higher CCT predicted faster RNFL thinning in this cohort. This finding could be due to treatment effect, as clinicians tend to treat patients with thinner CCT more aggressively based on their knowledge of the prognostic role of CCT, possibly leading to a confounding effect. Similarly, treatment effect might have affected the effect size of IOP.

In conclusion, a combination of lower SBP or DBP and higher IOP at baseline was associated with faster (worse) RNFL RoCs in this cohort of patients with central damage or moderate to advanced disease. Ongoing monitoring and management of BP and IOP in glaucoma patients might be a treatment strategy to help preserve RNFL thickness, potentially slowing disease progression and reducing the risk of vision loss. In order to consider the potential implications of BP management in this population, further research studies are needed to validate these findings and test the possible effect of any BP management strategies in glaucoma patients.

## Supplementary Material

1

2

Supplemental material available at www.ophthalmologyglaucoma.org.

## Figures and Tables

**Table 1.** Demographic and Clinical Characteristics of the Study Cohort at Baseline

Variable	Mean (SD)/n (%)	Median (IQR)
Follow-up duration (yrs)	4.3 (0.5)	-
Number of OCT scans	8.3 (1.4)	-
Age at baseline (yrs)	66.7 (8.6)	-
Sex (male/female), n (%)	42 (38.2%)/68 (61.8%)	-
Ethnicity/race, n (%)		-
Hispanic (all White)	13 (11.8%)	-
Non-Hispanic		-
White	57 (51.8%)	-
Asian	22 (20.0%)	-
Black	18 (16.4%)	-
Baseline diastolic blood pressure (mmHg)	81.9 (10.3)	-
Baseline systolic blood pressure (mmHg)	135.2 (18.1)	-
History of blood pressure medication at baseline, n (%)	40 (36.4%)	-
Hypertension (baseline), n (%)	41 (37.3%)	-
Diabetes mellitus (baseline), n (%)	17 (15.5%)	-
Baseline medicated intraocular pressure (mmHg)	12.6 (3.9)	-
Central corneal thickness (μm)	534.3 (40.2)	-
Axial length (mm)	24.6 (1.45)	-
Contrast sensitivity at 12 cycles per degree	3.9 (1.8)	-
Baseline 24-2 visual field mean deviation (dB)	−8.8 (6.0)	−6.8 (−12.4, −4.2)
Final 24-2 visual field mean deviation (dB)	−12.2 (7.0)	−11.2 (−16.2, −6.1)
Baseline pRNFL thickness (μm)	62.2 (13.6)	-

IQR = interquartile range; pRNFL = peripapillary retinal nerve fiber layer; SD = standard deviation.

**Table 2.** Results from the Multivariable Model Showing the Effect of Diastolic Blood Pressure and Its Interaction with IOP on RNFL Rate of Change

Variable	Posterior Mean	95% CrI	*P* Value
Age at baseline (/10 yrs)	0.008	(−0.046, 0.070)	0.394
Female sex	−0.263	(−0.398, 0.125)	**<** 0.001
Ethnicity			
White (reference)			
Black	−0.013	(−0.164, 0.128)	0.436
Hispanic	−0.345	(−0.559, −0.124)	**0.002**
Asian	−0.120	(−0.297, 0.022)	0.070
Hypertension	−0.004	(−0.125, 0.120)	0.470
History of blood pressure medication	−0.079	(−0.224, 0.031)	0.121
Diabetes mellitus	−0.234	(−0.414, −0.042)	**0.006**
Central corneal thickness (/10 μm)	−0.021	(−0.036, −0.005)	**0.005**
Axial length (/mm)	0.006	(−0.029, 0.049)	0.369
Contrast sensitivity at 12 cycles per degree	−0.056	(−0.093, −0.018)	**0.002**
24-2 visual field mean deviation (/dB)	−0.016	(−0.027, −0.005)	**0.002**
β-blocker use	−0.182	(−0.312, −0.045)	**0.004**
IOP (/mmHg)	−0.373	(−0.535, −0.207)	**< 0.001**
DBP (/10 mmHg)	−0.456	(−0.695, −0.207)	**< 0.001**
DBP (/10) × IOP interaction	0.043	(0.022, 0.063)	**< 0.001**

CrI = credible interval; DBP = diastolic blood pressure; IOP = intraocular pressure; RNFL = retinal nerve fiber layer. Boldface values represent statistically significant results.

**Table 3.** Results from the Multivariable Model Showing the Effect of Systolic Blood Pressure and Its Interaction with IOP on RNFL Rate of Change

Variable	Posterior Mean	95% CrI	*P* Value
Age at baseline (/10 yrs)	−0.001	(−0.065, 0.061)	0.494
Female sex	−0.243	(−0.383, −0.098)	**0.001**
Ethnicity			
White (reference)			
Black	0.005	(−0.142, 0.152)	0.470
Hispanic	−0.380	(−0.609, −0.130)	**0.002**
Asian	−0.141	(−0.315, 0.011)	0.046
Hypertension	−0.011	(−0.137, 0.108)	0.432
History of blood pressure medication	−0.085	(−0.233, 0.029)	0.104
Diabetes mellitus	−0.274	(−0.456, −0.089)	**0.002**
Central corneal thickness (/10 μm)	−0.020	(−0.036, −0.004)	**0.006**
Axial length (/mm)	0.007	(−0.028, 0.048)	0.367
Contrast sensitivity at 12 cycles per degree	−0.049	(−0.085, −0.011)	**0.004**
24-2 visual field mean deviation (/dB)	−0.016	(−0.028, −0.004)	**0.003**
β-blocker use	−0.176	(−0.305, −0.038)	**0.005**
IOP (/mmHg)	−0.221	(−0.363, −0.074)	**< 0.001**
SBP (/10 mmHg)	−0.132	(−0.253, −0.007)	**0.020**
SBP (/10) × IOP interaction	0.014	(0.003, 0.025)	**0.005**

CrI = credible interval; IOP = intraocular pressure; RNFL = retinal nerve fiber layer; SBP = systolic blood pressure. Boldface values represent statistically significant results.

## Abbreviations and Acronyms:

BP: blood pressure
CCT: central corneal thickness
DBP: diastolic blood pressure
IOP: intraocular pressure
MD: mean deviation
OAG: open-angle glaucoma
OPP: ocular perfusion pressure
RNFL: retinal nerve fiber layer
SBP: systolic blood pressure
SD: standard deviation

## References

[1] Nishida T, Moghimi S, Chang AC, Association of intraocular pressure with retinal nerve fiber layer thinning in patients with glaucoma. JAMA Ophthalmology. 2022;140:1209–1216. 36301523 10.1001/jamaophthalmol.2022.4462 PMC9614677

[2] Jammal AA, Berchuck SI, Mariottoni EB, Tanna AP, Blood pressure and glaucomatous progression in a large clinical population. Ophthalmology. 2022;129:161–170. 34474070 10.1016/j.ophtha.2021.08.021 PMC8792171

[3] De Moraes CG, Liebmann JM, Greenfield DS, Risk factors for visual field progression in the low-pressure glaucoma treatment study. Am J Ophthalmol. 2012;154:702–711. 22835512 10.1016/j.ajo.2012.04.015

[4] Leske MC, Heijl A, Hyman L, Predictors of long-term progression in the early manifest glaucoma trial. Ophthalmology. 2007;114:1965–1972. 17628686 10.1016/j.ophtha.2007.03.016

[5] Rao HL, Dasari S, Puttaiah NK, Optical microangiography and progressive retinal nerve fiber layer loss in primary open angle glaucoma. Am J Ophthalmol. 2022;233:171–179. 34320375 10.1016/j.ajo.2021.07.023 PMC8678163

[6] Collignon N, Dewe W, Guillaume S, Collignon-Brach J. Ambulatory blood pressure monitoring in glaucoma patients. The nocturnal systolic dip and its relationship with disease progression. Int Ophthalmol. 1998;22:19–25. 10090444 10.1023/a:1006113109864

[7] Choi J, Kim KH, Jeong J, Cho HS, Circadian fluctuation of mean ocular perfusion pressure is a consistent risk factor for normal-tension glaucoma. Invest Ophthalmol Vis Sci. 2007;48:104–111. 17197523 10.1167/iovs.06-0615

[8] Hayreh SS, Podhajsky P, Zimmerman MB. Role of nocturnal arterial hypotension in optic nerve head ischemic disorders. Ophthalmologica. 1999;213:76–96. 9885384 10.1159/000027399

[9] Tielsch JM, Katz J, Sommer A, Quigley HA, Hypertension, perfusion pressure, and primary open-angle glaucoma. A population-based assessment. Arch Ophthalmol. 1995;113:216–221. 7864755 10.1001/archopht.1995.01100020100038

[10] Graham SL, Drance SM. Nocturnal hypotension: role in glaucoma progression. Surv Ophthalmol. 1999;43:S10–S16. 10416743 10.1016/s0039-6257(99)00016-8

[11] Boland MV, Quigley HA. Risk factors and open-angle glaucoma: classification and application. J Glaucoma. 2007;16:406–418. 17571004 10.1097/IJG.0b013e31806540a1

[12] Mitchell P, Lee AJ, Rochtchina E, Wang JJ. Open-angle glaucoma and systemic hypertension: the blue mountains eye study. J Glaucoma. 2004;13:319–326. 15226661 10.1097/00061198-200408000-00010

[13] Drance SM, Sweeney VP, Morgan RW, Feldman F. Studies of factors involved in the production of low tension glaucoma. Arch Ophthalmol. 1973;89:457–465. 4706442 10.1001/archopht.1973.01000040459003

[14] Leske MC, Podgor MJ. Intraocular pressure, cardiovascular risk variables, and visual field defects. Am J Epidemiol. 1983;118:280–287. 6881131 10.1093/oxfordjournals.aje.a113634

[15] Quigley HA, West SK, Rodriguez J, Munoz B, The prevalence of glaucoma in a population-based study of Hispanic subjects: proyecto VER. Arch Ophthalmol. 2001;119:1819–1826. 11735794 10.1001/archopht.119.12.1819

[16] Chen DF, Wang C, Si Y, Natural history and risk factors for glaucoma progression in Chinese patients with normal-tension glaucoma. Invest Ophthalmol Vis Sci. 2024;65:28. 10.1167/iovs.65.3.28 PMC10959195 38506850

[17] Memarzadeh F, Ying-Lai M, Chung J, Azen SP, Los Angeles Latino eye study Group.Blood pressure, perfusion pressure, and open-angle glaucoma: the Los Angeles Latino eye study. Invest Ophthalmol Vis Sci. 2010;51:2872–2877. 20089880 10.1167/iovs.08-2956 PMC2891455

[18] Mohammadzadeh V, Su E, Mohammadi M, Association of blood pressure with rates of macular ganglion cell complex thinning in patients with glaucoma. JAMA Ophthalmol. 2023;141:251–257. 36757702 10.1001/jamaophthalmol.2022.6092 PMC9912170

[19] Lee K, Yang H, Kim JY, Seong GJ, Risk factors associated with structural progression in normal-tension glaucoma: intraocular pressure, systemic blood pressure, and myopia. Invest Ophthalmol Vis Sci. 2020;61:35. 10.1167/iovs.61.8.35 PMC7425752 32716503

[20] Caprioli J, Coleman AL. Discussion BFiG. Blood pressure, perfusion pressure, and glaucoma. Am J Ophthalmol. 2010;149:704–712. 20399924 10.1016/j.ajo.2010.01.018

[21] Charlson ME, de Moraes CG, Link A, Nocturnal systemic hypotension increases the risk of glaucoma progression. Ophthalmology. 2014;121:2004–2012. 24869467 10.1016/j.ophtha.2014.04.016 PMC4386594

[22] Leske MC, Wu SY, Hennis A, Honkanen R, Risk factors for incident open-angle glaucoma: the Barbados Eye Studies. Ophthalmology. 2008;115:85–93. 17629563 10.1016/j.ophtha.2007.03.017

[23] Dielemans I, Vingerling JR, Algra D, Hofman A, Primary open-angle glaucoma, intraocular pressure, and systemic blood pressure in the general elderly population. The Rotterdam Study. Ophthalmology. 1995;102:54–60. 7831042 10.1016/s0161-6420(95)31054-8

[24] Bonomi L, Marchini G, Marraffa M, Bernardi P, Vascular risk factors for primary open angle glaucoma: the Egna-Neumarkt Study. Ophthalmology. 2000;107:1287–1293. 10889099 10.1016/s0161-6420(00)00138-x

[25] Mohammadzadeh V, Su E, Shi L, Multivariate longitudinal modeling of macular ganglion cell complex: spatiotemporal correlations and patterns of longitudinal change. Ophthalmol Sci. 2022;2, 100187. 36245763 10.1016/j.xops.2022.100187 PMC9559093

[26] Mohammadzadeh V, Su E, Heydar ZS, Estimating ganglion cell complex rates of change with bayesian hierarchical models. Transl Vis Sci Technol. 2021;10:15. 10.1167/tvst.10.4.15 PMC8054624 34003991

[27] Carvalho CM, Polson NG, Scott JG. Handling sparsity via the horseshoe. In: Proceedings of the Twelfth International Conference on Artificial Intelligence and Statistics. Florida: Clearwater Beach; 2009:73–80; 5.

[28] Carvalho CM, Polson NG, Scott JG. The horseshoe estimator for sparse signals. Biometrika. 2010;97:465–480.

[29] Plummer M JAGS: a Program for analysis of bayesian graphical models using gibbs sampling. Proceedings of the 3rd international workshop on distributed statistical computing (DSC 2003), Vienna Austria. http://www.r-project.org/conferences/DSC-2003/. Accessed March 20, 2022.

[30] R Core Team. R: A Language and Environment for Statistical Computing. Vienna, Austria: R Foundation for Statistical Computing; 2023.

[31] Su YS, Yajima M. R2jags: Using R to Run ’JAGS’. R package version 0.7-1 2021.

[32] Leske MC. Ocular perfusion pressure and glaucoma: clinical trial and epidemiologic findings. Curr Opin Ophthalmol. 2009;20:73–78. 19240538 10.1097/ICU.0b013e32831eef82 PMC2662722

[33] Levine RM, Yang A, Brahma V, Martone JF. Management of blood pressure in patients with glaucoma. Curr Cardiol Rep. 2017;19:109. 28929290 10.1007/s11886-017-0927-x

[34] Lee EB, Hu W, Singh K, Wang SY. The association among blood pressure, blood pressure medications, and glaucoma in a Nationwide electronic health records database. Ophthalmology. 2022;129:276–284. 34688700 10.1016/j.ophtha.2021.10.018 PMC8863625

[35] Topouzis F, Wilson MR, Harris A, Risk factors for primary open-angle glaucoma and pseudoexfoliative glaucoma in the Thessaloniki eye study. Am J Ophthalmol. 2011;152:219–228.e1. 21664597 10.1016/j.ajo.2011.01.032

[36] Jammal AA, Thompson AC, Mariottoni EB, Impact of intraocular pressure control on rates of retinal nerve fiber layer loss in a large clinical population. Ophthalmology. 2021;128(1):48–57. 32579892 10.1016/j.ophtha.2020.06.027 PMC7750282

[37] Chauhan BC. Endothelin and its potential role in glaucoma. Can J Ophthalmol. 2008;43:356–360. 18493277 10.3129/i08-060

[38] Jin SW, Seo HR, Rho SS, Rho SH. The effects of nocturnal dip and blood pressure variability on paracentral scotoma in early open-angle glaucoma. Semin Ophthalmol. 2017;32:504–510. 27128963 10.3109/08820538.2015.1123733

[39] Pappelis K, Jansonius NM. U-shaped effect of blood pressure on structural OCT metrics and retinal perfusion in ophthalmologically healthy subjects. Invest Ophthalmol Vis Sci. 2021;62:5. 10.1167/iovs.62.12.5 PMC8434757 34499704

[40] Rajasundaram S, Segrè AV, Gill D, Independent effects of blood pressure on intraocular pressure and retinal ganglion cell degeneration: a mendelian randomization study. Invest Ophthalmol Vis Sci. 2024;65:35. 10.1167/iovs.65.8.35 PMC11262474 39028976

