# Supplementary material for: Association of Blood Pressure and Retinal Nerve Fiber Layer Rates of Thinning in Patients with Moderate to Advanced Glaucoma
Source: Ophthalmol Glaucoma. Author manuscript; Available in PMC 2026 Apr 11. (PMC13070006; doi:10.1016/j.ogla.2024.12.009)

**Figure S1**. Estimated RNFL slopes pooled across 12 sectors plotted against diastolic blood pressure (DBP) at baseline.


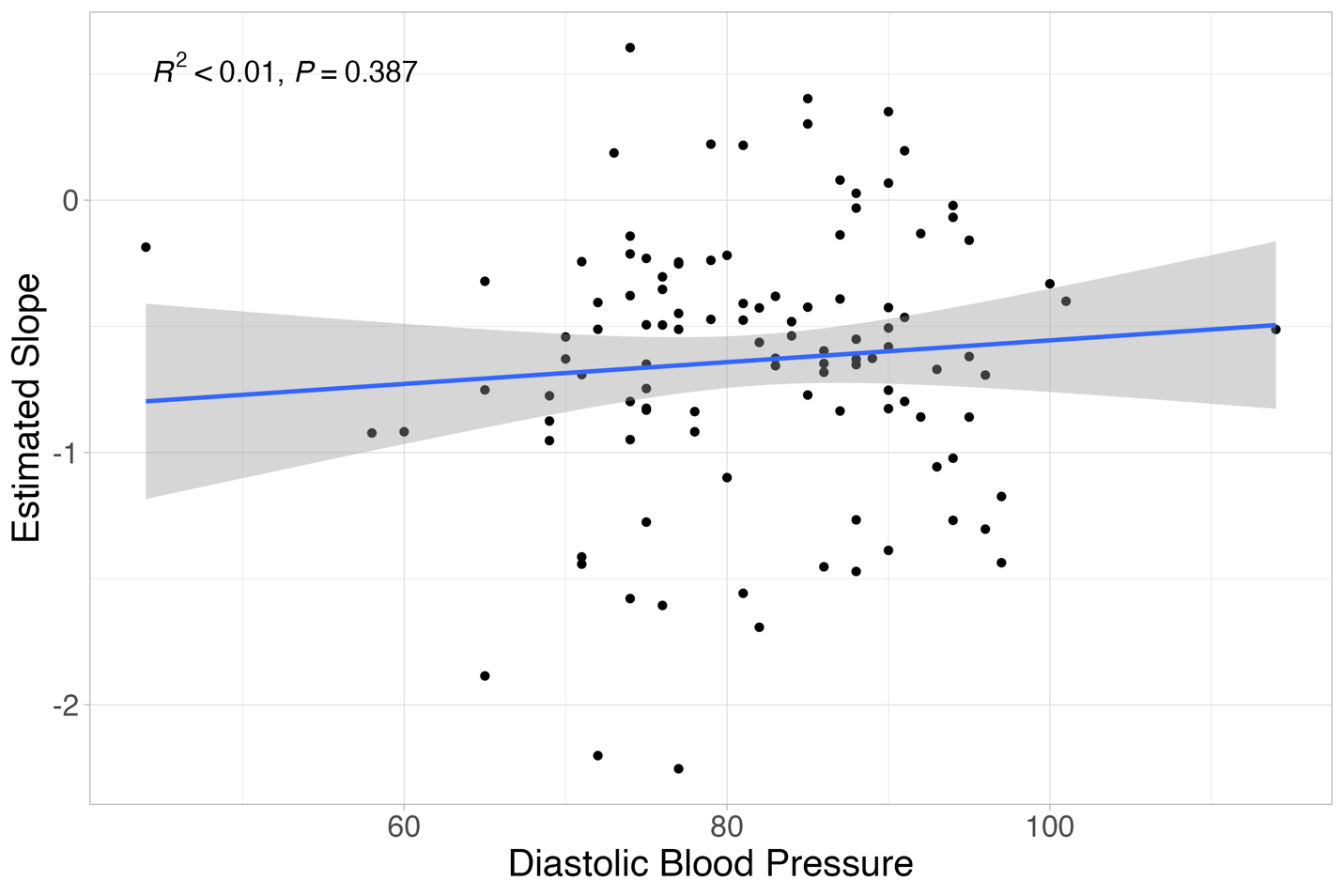


**Figure S2**. Estimated RNFL slopes pooled across 12 sectors plotted against systolic blood pressure (SBP) at baseline.


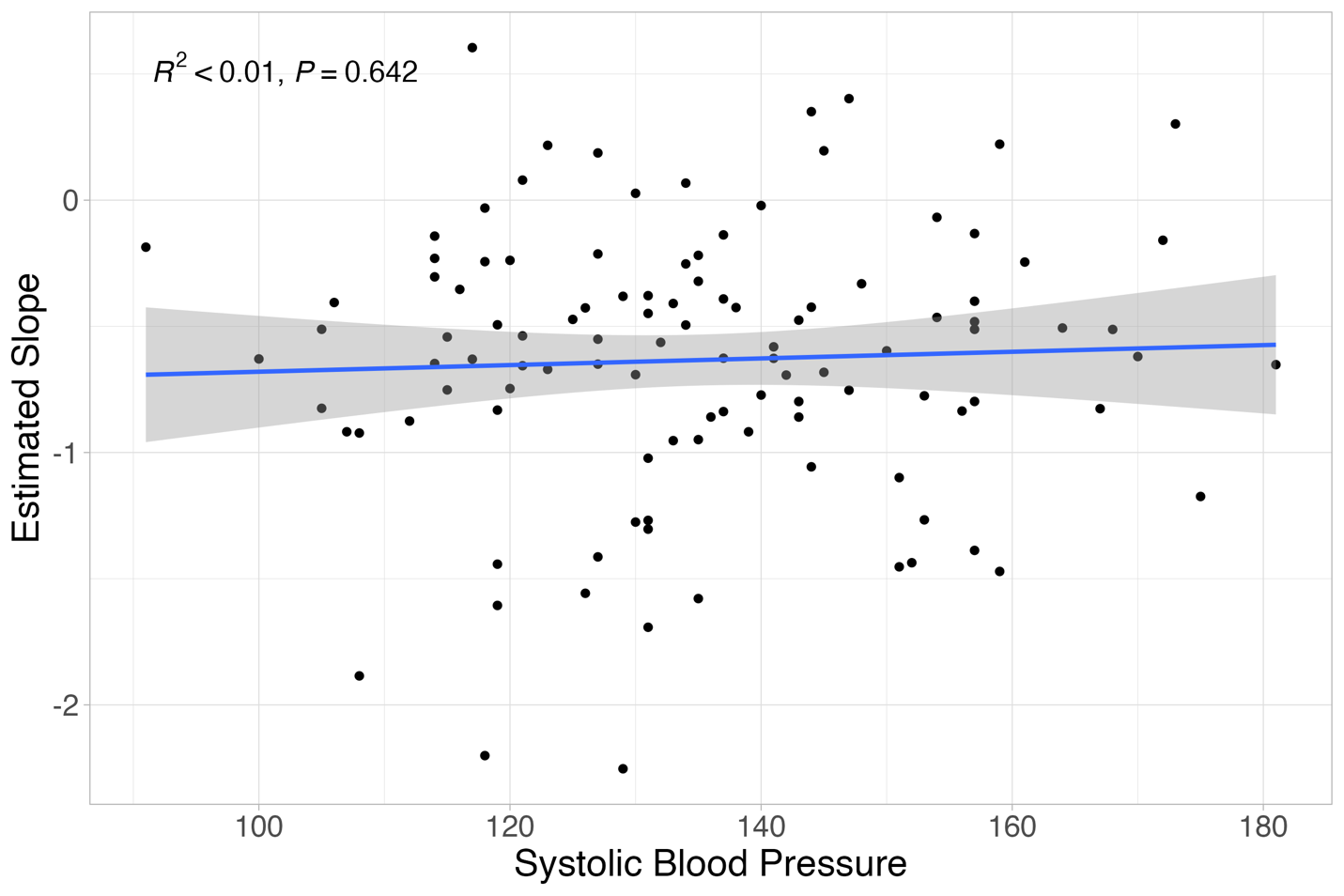


**Figure S3**. Estimated RNFL slopes pooled across 12 sectors plotted against intraocular pressure (IOP) at baseline.


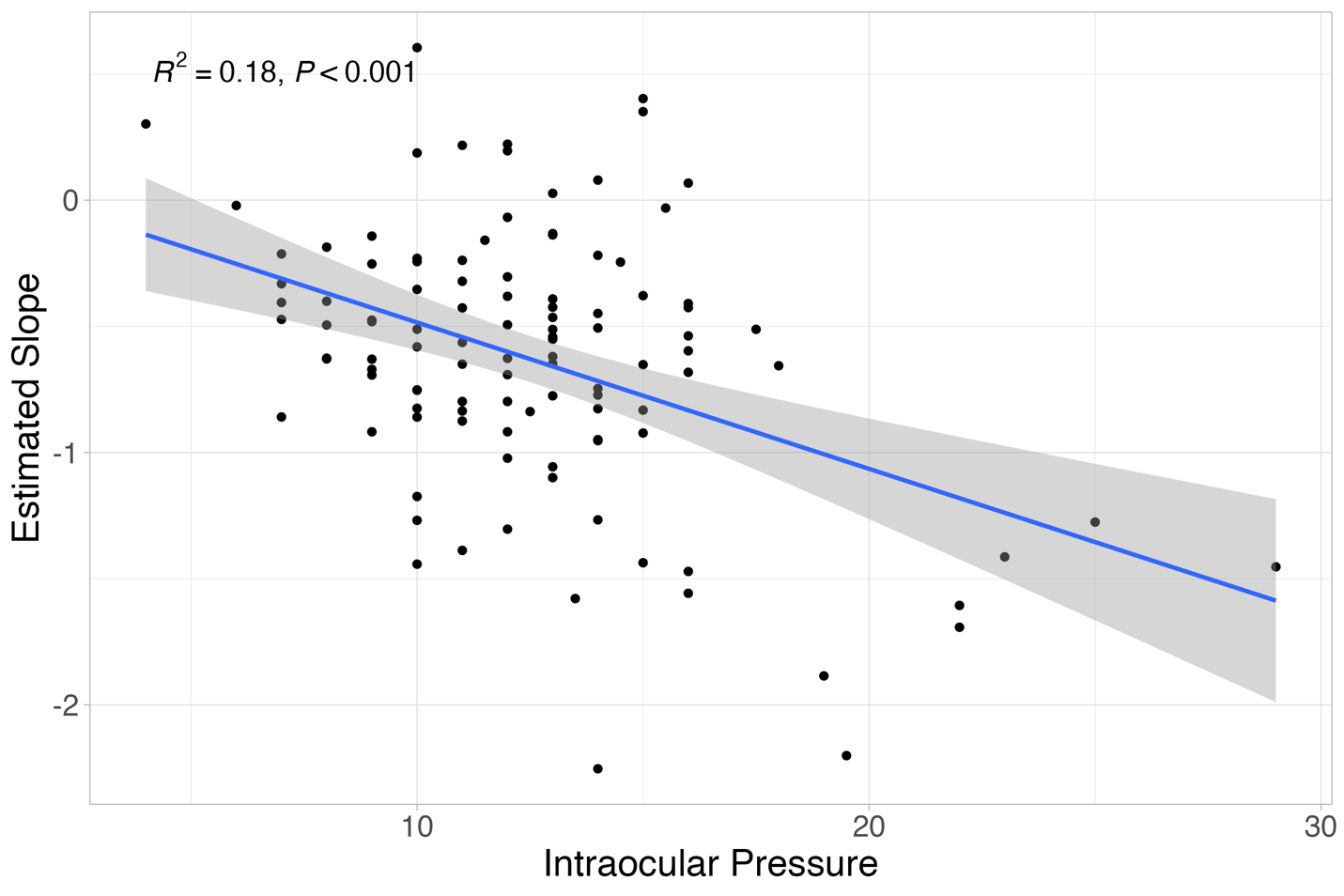


**Figure S4**. Estimated RNFL slopes pooled across 12 sectors plotted against age at baseline.


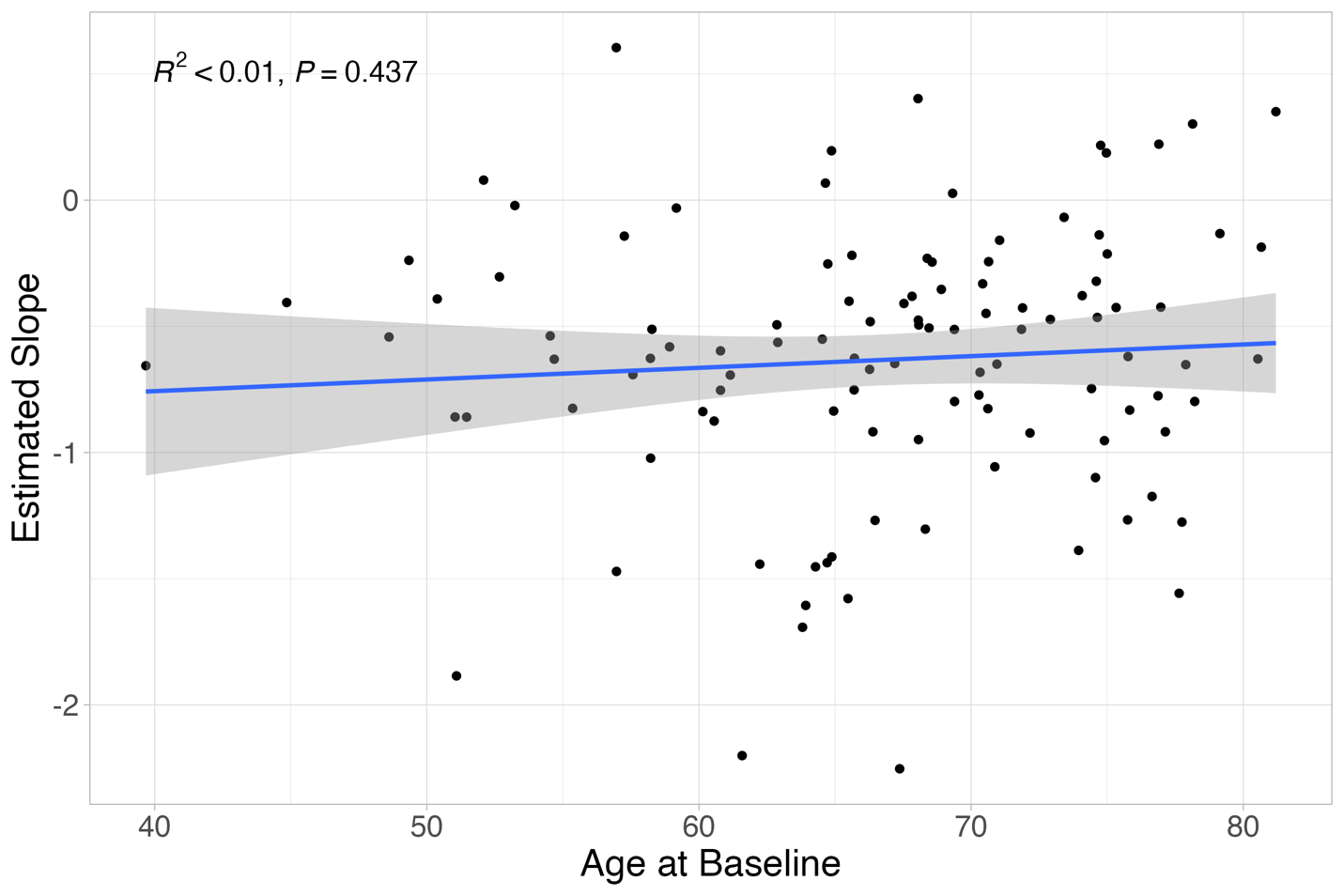

Supplement: 1 [file NIHMS2159653-supplement-1.docx]
