# Supplementary material for: Association of Blood Pressure and Retinal Nerve Fiber Layer Rates of Thinning in Patients with Moderate to Advanced Glaucoma
Source: Ophthalmol Glaucoma. Author manuscript; Available in PMC 2026 Apr 11. (PMC13070006; doi:10.1016/j.ogla.2024.12.009)
Supplement: 2 [file NIHMS2159653-supplement-2.docx]

**Supplementary Online Content**

Figueroa J, Su E, Mohammadzadeh V, et al. Association of Blood Pressure and RNFL Rates of Thinning in Patients with Moderate to Advanced Glaucoma

**Supplementary Methods.** Bayesian Hierarchical Longitudinal Model with Random Intercepts, Slopes, and Residual Variances

**Table S1.** Results from the univariate models showing the association of each covariate on the RNFL intercepts.

**Table S2.** Results from the univariate models showing the association of each covariate on the RNFL rates of change.

**Table S3.** Results from the multivariable model showing the effect of diastolic blood pressure and its interaction with IOP on the RNFL intercepts.

**Table S4.** Results from the multivariable model showing the effect of systolic blood pressure and its interaction with IOP on the RNFL intercepts.

**Table S5.** Posterior means, 95% credible intervals (CrI), and 1-sided p-values of estimated slopes for hypothetical subjects at the 10^th^ and 90^th^ quantiles for DBP and IOP.

**Table S6.** Posterior means, 95% credible intervals (CrI), and 1-sided p-values of estimated slopes for hypothetical subjects at the 10^th^ and 90^th^ quantiles for SBP and IOP.

**Table S7.** Posterior means, 95% credible intervals (CrI), and 1-sided p-values of differences (group 1 – group 2) in estimated slopes between hypothetical subjects at the 10^th^ and 90^th^ quantiles for DBP and IOP.

**Table S8.** Posterior means, 95% credible intervals (CrI), and 1-sided p-values of differences (group 1 – group 2) in estimated slopes between hypothetical subjects at the 10^th^ and 90^th^ quantiles for SBP and IOP.

**Figure S1.** Results from estimated RNFL slopes pooled across 12 sectors plotted against diastolic blood pressure (DBP) at baseline.

**Figure S2.** Results from the estimated RNFL slopes pooled across 12 sectors plotted against systolic blood pressure (SBP) at baseline.

**Figure S3.** Results from the estimated RNFL slopes pooled across 12 sectors plotted against intraocular pressure (IOP) at baseline.

**Figure S4.** Results from the estimated RNFL slopes pooled across 12 sectors plotted against age at baseline.

**Methods**

*Details on the Outlier Removal Algorithm*

As described previously, we applied a semi-automated algorithm to identify and remove retinal nerve fiber layer (RNFL) outliers. Prior to applying the removal algorithm, we removed all RNFL measurements of 0$\mu$m, indicative of a reporting error. Let $y_{ijk}$ denote the RNFL thickness measured on subject $i$ at time $t_{ij}$ in sector $k$ for $k=1,\ldots, 12$. Time since baseline $t_{ij}$ is measured in years, where the first visit $j=1$ for all participants is at $t_{i1}=0$ years. For each subject’s longitudinal data in each sector, we calculated consecutive visit absolute differences ${|y}_{ijk}-y_{i\left( j-1 \right)k}|$ and consecutive visit absolute centered slopes ${| ((y}_{ijk}-y_{i\left( j-1 \right)k})/(t_{ij}-t_{i\left( j-1 \right)}))+0.71|$; slopes were centered around −0.71$\mu$m/year, the mean of the pooled set of slopes from all pairs of consecutive visits across all participants and sectors. We flagged absolute centered slopes greater than 37 $\mu$m/year. This value was chosen to identify approximately 1.0% of the observations as possible outliers. The flagged slope identified two consecutive points as candidates for removal. We calculated the sum of the absolute visit differences $\sum_{j=2}^{n_{ik}} |y_{ijk}- y_{i\left( j-1 \right)k}|$ for each sector in each participant and further considered the candidate that caused the largest decrease in the sum of the absolute visit differences. If removing the candidate resulted in a replacement absolute slope that was not one-half or less of the original flagged slope, we did not remove the observation; otherwise, we removed the candidate. If an observation was removed, we applied the same algorithm to the reduced data set to see if another observation from the same sector should be removed as well. For each curve, if 2 or more points were identified as outliers, we removed the entire curve. This resulted in removing 1.1% of the observations as outliers.

*Bayesian Hierarchical Longitudinal Model with Random Intercepts, Slopes, and Residual Variances*

For this study, we fit longitudinal retinal nerve fiber layer (RNFL) measurements from all participants and sectors together in a Bayesian hierarchical random-effects model. We adapt our previous Bayesian hierarchical model for use on RNFL sectors.^1^ In each sector, our model has 7 interpretable sector-level population parameters, the (i) population intercept $\alpha_{0k}$, (ii) standard deviation (SD) of the random intercepts $D_{00k}^{1/2}$, (iii) population slope $\alpha_{1k}$, (iv) SD of the random slopes $D_{11k}^{1/2}$, (v) correlation $\rho_{k}$ between random intercepts and slopes, (vi) mean $\sigma_{mk}$ of the random residual SDs, and (vii) SD $\sigma_{sk}$ of the random residual SDs. We transformed the random intercepts and slopes correlation $\rho_{k}$ and variance $D_{11k}$ of the random slopes to the regression coefficient $\gamma_{k}=\rho_{k}D_{11k}^{1/2}D_{00k}^{-1/2}$ of the random slopes given the random intercept and the remaining slope variance $D_{11.0k}=\left( 1-\rho_{k}^{2} \right)D_{11k}$ of the random slopes (variance of the random slopes adjusted for the random intercepts). We log transformed the random intercept and remaining slope variances $D_{00k}$, $D_{11.0k}$, and the population mean $\sigma_{mk}$ and SD $\sigma_{sk}$ of the random residual SDs. We term these 7 sector parameters (or their transformations) population parameters. The 7 transformed parameters in sector $k=1,\ldots, 12$ were given hierarchical normal priors with unknown global mean and variance. Three parameters, $\alpha_{0k},\log D_{00k},\log D_{11.0k}$, were given a joint multivariate normal prior, the other four parameters were given independent normal priors.

For sector $k$, $\alpha_{0k}$ is the population average intercept at baseline $t_{i1}=0$; $\alpha_{1k}$ is the population average slope; $\beta_{0ik}$ is the $i$th subject’s random intercept: the unknown difference between subject $i$'s intercept and the population intercept $\alpha_{0k}$; $\beta_{1ik}$ is the $i$th subject’s random slope: the unknown difference between subject $i$'s slope and $\alpha_{1k}$; and the residual variance for subject $i$ is $\sigma_{\mathrm{ik}}^{2}$. For subject $i$, $x_{i}=\left( x_{i1},\ldots, x_{\mathrm{iQ}} \right)^{'}$ are the Q standardized covariates of interest; $\eta_{0}=\left( \eta_{01}, \ldots, \eta_{0Q} \right)'$ is the vector of regression coefficients for the covariate effects on the intercepts; and $\eta_{1}=\left( \eta_{11}, \ldots, \eta_{1Q} \right)'$ is the vector of regression coefficients for the covariate effects on the slopes. We standardized all covariates by subtracting the covariate sample mean $m_{p}$ and then dividing by the covariate sample SD $s_{p}$. This includes standardizing indicator variables for the three race categories (with White as the reference category) and for the indicator of female sex. We report inferences for coefficients by transforming coefficients back to being coefficients of unstandardized coefficients $\eta_{p}^{*}=\eta_{p}/s_{p}$ in the absence of an interaction. Covariates that were indicator variables were standardized as $x_{i}^{*}=(x_{i}-p)/[\mathrm{sqrt}(p(1-p))]$ where $p$ is the mean of $x_{i}$ over subjects, so that the $x_{i}^{*}$ have mean 0 and standard deviation (SD) 1. This makes all predictors to have mean zero and SD 1 before putting a prior on the regression coefficients. Priors on the coefficients of these standardized predictors are identical, so that all predictors are given *a* *priori* equal odds of being significant or not and of having positive or negative effects. In reporting results, we back transformed the coefficients and reported the coefficients of the unstandardized predictors.

For models with interactions between two covariates, we multiply both standardized covariates together, and do not further standardize the interaction term. To transform standardized coefficients of covariates labeled 1 and 2, and the interaction term between covariate 1 and 2 back to being unstandardized coefficients, we use the following formulas

$$\eta_{1}^{*}=\left( \frac{\eta_{1}}{s_{1}} \right)-\left( \frac{m_{2}}{s_{1}s_{2}} \right)\eta_{1\times2}$$

$$\eta_{2}^{*}=\left( \frac{\eta_{2}}{s_{2}} \right)-\left( \frac{m_{1}}{s_{1}s_{2}} \right)\eta_{1\times2}$$

$$\eta_{1\times2}^{*}=\left( \frac{\eta_{1x2}}{s_{1}s_{2}} \right) .$$

Let $N\left( a,b^{2} \right)$ be a normal random variable with mean $a$ and variance $b^{2}$;$IG(a,b)$ be an inverse gamma random variable with shape parameter $a$, scale parameter $b$, and mean $b/(a-1)$ for $a>1$; $C^{+}\left( a,b \right)$ be a half-Cauchy random variable (a Cauchy or t with 1 degree of freedom restricted to the positive real line) with location $a$ and scale $b$; and $\mathrm{Wish}(V,n)$ for Wishart distribution with inverse scale matrix $V$, degrees of freedom $n$, and mean $(n{V)}^{-1}$. For the univariable models, we fit a separate model for each covariate ($Q=1$). The full multivariable model is

$$y_{\mathrm{ijk}}={\alpha_{0k}+ \alpha_{1k}t_{\mathrm{ij}}+\beta}_{0ik}+\beta_{1ik}t_{\mathrm{ij}} +\eta_{0}^{'}x_{i}+ \eta_{1}^{'}x_{i}t_{\mathrm{ij}}+\epsilon_{\mathrm{ijk}}$$

$$\epsilon_{\mathrm{ijk}}|\sigma_{\mathrm{ik}}^{2} \sim N(0, \sigma_{\mathrm{ik}}^{2})$$

$$\beta_{0ik}|D_{00k} \sim N(0,D_{00k})$$

$$\beta_{1ik}|{\gamma_{k},\beta_{0ik},D}_{11.0k} \sim N(\gamma_{k}\beta_{0ik},D_{11.0k})$$

$$\log\sigma_{\mathrm{ik}}| \sigma_{\mathrm{mk}},\sigma_{\mathrm{sk}}\sim N(\mu^{*},\sigma^{*2})$$

$$\mu^{*}=2 \log\sigma_{\mathrm{mk}}-0.5 \log{(e}^{2\log\sigma_{\mathrm{mk}}}+e^{2\sigma_{\mathrm{sk}}})$$

$$\sigma^{*2}=\log e^{2(log \sigma_{\mathrm{sk}}-\log\sigma_{\mathrm{mk}})+1}$$

$$\left( \alpha_{0k},\log D_{00k},\log D_{11.0k} \right)^{'} |\left( \mu_{1}, \mu_{2},\mu_{4} \right)^{'}, \Sigma\sim N(\left( \mu_{1}, \mu_{2},\mu_{4} \right)^{'}, \Sigma)$$

$$\alpha_{1k}|\mu_{3}, \sigma_{3}^{2} \sim N(\mu_{3}, \sigma_{3}^{2})$$

$$\gamma_{k}|\mu_{5}, \sigma_{5}^{2} \sim N(\mu_{5}, \sigma_{5}^{2})$$

$$\log\sigma_{\mathrm{mk}}|\mu_{6}, \sigma_{6}^{2} \sim N(\mu_{6}, \sigma_{6}^{2})$$

$$\log\sigma_{\mathrm{sk}}|\mu_{7}, \sigma_{7}^{2} \sim N(\mu_{7}, \sigma_{7}^{2})$$

Regression coefficients have horseshoe priors ^2,3^

$$\eta_{0p}|\lambda_{0p},\tau_{0} \sim N(0, \lambda_{0p}^{2}\tau_{0}^{2})$$

$$\eta_{1p}|\lambda_{1p},\tau_{1} \sim N(0, \lambda_{1p}^{2}\tau_{1}^{2})$$

for $p=1,\ldots,Q$, $N(0, \lambda_{p}^{2}\tau^{2})$, with separate $\lambda_{0p}$ and $\lambda_{1p}$ for covariate $p$ for the intercept and slope respectively, and global parameters $\tau_{0}$ and $\tau_{1}$, where $\lambda_{p}$ and $\tau$ are a priori distributed as independent half-Cauchy random variables with location zero and scale parameter 1.

Matrix $D_{k}$ is a $2x2$ variance-covariance matrix of the random intercepts and slopes with elements

$$D_{k}=\left( \begin{aligned} D_{00k} D_{01k} \\ D_{10k}D_{11k} \end{aligned} \right)$$

and $D_{11.0k}=D_{11k}-D_{10k}D_{00k}^{-1}D_{01k}$ is the variance of the conditional distribution of $\beta_{1ik}|\beta_{0ik}$. The correlation between the random intercepts and slopes is

$$\rho_{k}= \frac{D_{01k}}{\left( D_{00k} D_{11k} \right)^{1/2}}=\gamma_{k}\sqrt{\frac{D_{00k}}{D_{11k}}} .$$

The priors are

$$\mu_{1} \sim N(90, 400)$$

$$\mu_{2} \sim N(5.4161, 0.804719)$$

$$\mu_{3} \sim N(-0.8, 0.36)$$

$$\mu_{4} \sim N(-0.4462871, 0.804719)$$

$$\mu_{5} \sim N(0, 9e-04)$$

$$\mu_{6} \sim N(0.7, 0.09)$$

$$\mu_{7} \sim N(-0.25, 0.09)$$

$$\Sigma^{-1} \sim Wish(5V, 5)$$

$$V=\left( \begin{matrix} 135 & 0 & 0 \\ 0 & 0.15 & 0 \\ 0 & 0 & 0.384 \end{matrix} \right)$$

$$\sigma_{3}^{2} \sim IG(2.5, 0.1666667)$$

$$\sigma_{5}^{2} \sim IG(2.5, 0.00135)$$

$$\sigma_{6}^{2} \sim IG(2.5, 0.06)$$

$$\sigma_{7}^{2} \sim IG(2.5, 0.135)$$

$$\lambda_{0p} \sim C^{+}(0, 1)$$

$$\lambda_{1p} \sim C^{+}\left( 0, 1 \right)$$

$$\tau_{0} \sim C^{+}\left( 0, 1 \right)$$

$\tau_{1} \sim C^{+}(0, 1)$.

**Table S1.** Results from the univariate models showing the influence of each covariate on the RNFL intercepts.

| *Variable* | *Posterior Mean* | *95% CI* | *p-value* |
| --- | --- | --- | --- |
| Age at Baseline (/10 years) | –0.094 | (–0.954, 0.447) | 0.412 |
| Female Sex | 4.351 | (2.096, 6.557) | **<0.001** |
| Ethnicity |  |  |  |
| White (reference) |  |  |  |
| African American | –0.538 | (–3.107, 0.749) | 0.305 |
| Hispanic | 0.536 | (–1.004, 3.350) | 0.326 |
| Asian | 4.832 | (1.505, 7.715) | **0.003** |
| Hypertension | 2.482 | (–0.018, 4.914) | 0.035 |
| History of BP Medication | 0.892 | (–0.329, 3.348) | 0.197 |
| Diabetes Mellitus | -0.358 | (–2.734, 1.094) | 0.356 |
| Central Corneal Thickness (/10 μm) | 0.011 | (–0.134, 0.185) | 0.448 |
| Axial Length (/mm) | –0.123 | (–0.776, 0.234) | 0.326 |
| Contrast Sensitivity at 12 cycles per degree | –0.260 | (–0.897, 0.115) | 0.170 |
| Mean Deviation 24–2 (/dB) | 1.161 | (1.001, 1.326) | **<0.001** |
| Betablocker Use | –0.515 | (–2.522, 0.597) | 0.269 |
| Intraocular Pressure (/mmHg) | 0.143 | (–0.040, 0.440) | 0.135 |
| Diastolic Blood Pressure (/10 mmHg) | 0.122 | (–0.383, 0.957) | 0.361 |
| Systolic Blood Pressure (/10 mmHg) | –0.144 | (–0.729, 0.129) | 0.274 |

**Table S2.** Results from the univariate models showing the association of each covariate with the RNFL rates of change.

| *Variable* | *Posterior Mean* | *95% CI* | *p-value* |
| --- | --- | --- | --- |
| Age at Baseline (/10 years) | 0.021 | (–0.029, 0.089) | 0.246 |
| Female Sex | –0.222 | (–0.353, –0.089) | **0.001** |
| Ethnicity |  |  |  |
| White (reference) |  |  |  |
| African American | 0.053 | (–0.088, 0.226) | 0.266 |
| Hispanic | –0.231 | (–0.431, –0.018) | **0.014** |
| Asian | –0.178 | (–0.345, –0.005) | **0.019** |
| Hypertension | –0.073 | (–0.207, 0.037) | 0.125 |
| History of BP Medication | –0.045 | (–0.172, 0.052) | 0.225 |
| Diabetes Mellitus | –0.250 | (–0.413, –0.076) | **0.002** |
| Central Corneal Thickness (/10 μm) | –0.020 | (–0.035, –0.004) | **0.005** |
| Axial Length (/mm) | 0.052 | (0.004, 0.098) | **0.014** |
| Contrast Sensitivity at 12 cycles per degree | –0.054 | (–0.088, –0.019) | **0.002** |
| Mean Deviation 24–2 (/dB) | –0.019 | (–0.029, –0.008) | **<0.001** |
| Betablocker Use | –0.285 | (–0.402, –0.169) | **<0.001** |
| Intraocular Pressure (/mmHg) | –0.048 | (–0.064, –0.032) | **<0.001** |
| Diastolic Blood Pressure (/10 mmHg) | 0.045 | (–0.007, 0.107) | 0.069 |
| Systolic Blood Pressure (/10 mmHg) | 0.013 | (–0.012, 0.046) | 0.206 |

**Table S3.** Results from the multivariable model showing the effect of diastolic blood pressure and its interaction with IOP on the RNFL intercepts.

| *Variable* | *Posterior Mean* | *95% CI* | *p-value* |
| --- | --- | --- | --- |
| Age at Baseline (/10 years) | –0.102 | (–1.089, 0.696) | 0.418 |
| Female Sex | 4.664 | (2.391, 6.863) | **<0.001** |
| Ethnicity |  |  |  |
| White (reference) |  |  |  |
| African American | –0.172 | (–2.287, 1.621) | 0.441 |
| Hispanic | 1.436 | (–0.744, 4.728) | 0.155 |
| Asian | 3.311 | (0.263, 6.192) | **0.011** |
| Hypertension | 2.436 | (0.000, 5.078) | **0.025** |
| History of BP Medication | –0.248 | (–2.204, 1.377) | 0.393 |
| Diabetes Mellitus | 0.618 | (–1.103, 3.057) | 0.284 |
| Central Corneal Thickness (/10 μm) | 0.133 | (–0.045, 0.388) | 0.120 |
| Axial Length (/mm) | –0.507 | (–1.327, 0.088) | 0.087 |
| Contrast Sensitivity at 12 cycles per degree | –0.605 | (–1.228, 0.001) | 0.026 |
| Mean Deviation 24–2 (/dB) | 1.174 | (1.002, 1.344) | **<0.001** |
| Betablocker Use | –0.473 | (–2.266, 0.795) | 0.273 |
| Intraocular Pressure (/mmHg) | 0.789 | (–0.741, 3.173) | 0.220 |
| Diastolic Blood Pressure (/10 mmHg) | 1.623 | (–0.751, 5.168) | 0.110 |
| DBP (/10) x IOP Interaction | –0.099 | (–0.390, 0.087) | 0.207 |

**Table S4.** Results from the multivariable model showing the effect of systolic blood pressure and its interaction with IOP on the RNFL intercepts.

| *Variable* | *Posterior Mean* | *95% CI* | *p-value* |
| --- | --- | --- | --- |
| Age at Baseline (/10 years) | –0.127 | (–1.095, 0.642) | 0.392 |
| Female Sex | 4.610 | (2.301, 6.824) | **<0.001** |
| Ethnicity |  |  |  |
| White (reference) |  |  |  |
| African American | –0.083 | (–2.034, 1.754) | 0.465 |
| Hispanic | 1.551 | (–0.731, 5.022) | 0.145 |
| Asian | 3.272 | (0.118, 6.181) | **0.015** |
| Hypertension | 2.480 | (0.005, 5.184) | **0.024** |
| History of BP Medication | –0.287 | (–2.316, 1.264) | 0.380 |
| Diabetes Mellitus | 0.590 | (–1.125, 3.020) | 0.290 |
| Central Corneal Thickness (/10 μm) | 0.118 | (–0.054, 0.373) | 0.144 |
| Axial Length (/mm) | –0.503 | (–1.330, 0.084) | 0.085 |
| Contrast Sensitivity at 12 cycles per degree | –0.528 | (–1.144, 0.016) | 0.038 |
| Mean Deviation 24–2 (/dB) | 1.175 | (1.005, 1.341) | **<0.001** |
| Betablocker Use | –0.527 | (–2.381, 0.718) | 0.256 |
| Intraocular Pressure (/mmHg) | 0.024 | (–1.290, 1.420) | 0.499 |
| Systolic Blood Pressure (/10 mmHg) | 0.042 | (–1.170, 1.324) | 0.478 |
| SBP (/10) x IOP Interaction | –0.003 | (-0.107, 0.094) | 0.477 |

**Additional Tables of Estimated Slopes for Hypothetical Subjects**

**Table S5**. Posterior means, 95% credible intervals (CrI), and 1-sided p values of estimated slopes for hypothetical subjects at the 10^th^ and 90^th^ quantiles for DBP and IOP.

| **DBP** | **IOP** | **Posterior Mean** | **95% CrI** | **p-value** |
| --- | --- | --- | --- | --- |
| 10^th^ | 10^th^ | −0.010 | (−0.251, 0.222) | 0.470 |
| 10^th^ | 90^th^ | −0.554 | (−0.793, −0.319) | **<0.001** |
| 90^th^ | 10^th^ | −0.268 | (−0.500, −0.041) | **0.009** |
| 90^th^ | 90^th^ | −0.017 | (−0.305, 0.258) | 0.463 |

**Table S6**. Posterior means, 95% credible intervals (CrI), and 1-sided p values of estimated slopes for hypothetical subjects at the 10^th^ and 90^th^ quantiles for SBP and IOP.

| **SBP** | **IOP** | **Posterior Mean** | **95% CrI** | **p-value** |
| --- | --- | --- | --- | --- |
| 10^th^ | 10^th^ | −0.082 | (−0.311, 0.142) | 0.239 |
| 10^th^ | 90^th^ | −0.539 | (−0.784, −0.301) | **<0.001** |
| 90^th^ | 10^th^ | −0.156 | (−0.404, 0.085) | 0.103 |
| 90^th^ | 90^th^ | −0.118 | (−0.422, 0.176) | 0.215 |

**Table S7**. Posterior means, 95% credible intervals (CrI), and 1-sided p values of differences (group 1 – group 2) in estimated slopes between hypothetical subjects at the 10^th^ and 90^th^ quantiles for DBP and IOP.

| **Group 1** | | **Group 2** | | **Difference** | **95% CrI** | **p-value** |
| --- | --- | --- | --- | --- | --- | --- |
| **DBP** | **IOP** | **DBP** | **IOP** |  |  |  |
| 10 | 90 | 10 | 10 | −0.544 | (−0.758, −0.329) | **<0.001** |
| 10 | 90 | 90 | 10 | −0.286 | (−0.493, −0.077) | **0.005** |
| 10 | 90 | 90 | 90 | −0.537 | (−0.785, −0.282) | **<0.001** |
| 90 | 10 | 10 | 10 | −0.258 | (−0.484, −0.027) | **0.015** |
| 90 | 10 | 90 | 90 | −0.251 | (−0.515, 0.019) | 0.034 |
| 90 | 90 | 10 | 10 | −0.006 | (−0.236, 0.218) | 0.479 |

**Table S8**. Posterior means, 95% credible intervals (CrI), and 1-sided p values of differences (group 1 – group 2) in estimated slopes between hypothetical subjects at the 10^th^ and 90^th^ quantiles for SBP and IOP.

| **Group 1** | | **Group 2** | | **Difference** | **95% CrI** | **p-value** |
| --- | --- | --- | --- | --- | --- | --- |
| **SBP** | **IOP** | **SBP** | **IOP** |  |  |  |
| 10 | 90 | 10 | 10 | −0.458 | (−0.686, −0.229) | **<0.001** |
| 10 | 90 | 90 | 10 | −0.383 | (−0.613, −0.142) | **<0.001** |
| 10 | 90 | 90 | 90 | −0.421 | (−0.702, −0.127) | **0.001** |
| 90 | 10 | 10 | 10 | −0.075 | (−0.293, 0.146) | 0.251 |
| 90 | 10 | 90 | 90 | −0.038 | (−0.284, 0.215) | 0.377 |
| 90 | 90 | 10 | 10 | −0.037 | (−0.271, 0.196) | 0.388 |

**References**

1. Mohammadzadeh V, Su E, Mohammadi M, et al. Association of Blood Pressure With Rates of Macular Ganglion Cell Complex Thinning in Patients With Glaucoma. *JAMA Ophthalmol*. Mar 01 2023;141(3):251-257. doi:10.1001/jamaophthalmol.2022.6092

2. Carvalho CM, Polson NG, Scott JG. Handling sparsity via the horseshoe. In: *Proceedings of the Twelfth International Conference on Artificial Intelligence and Statistics*. Clearwater Beach, Florida. *PMLR*. 2009;5:73-80.; April 16-18, 2009.

3. Carvalho CM, Polson NG, Scott JG. The horseshoe estimator for sparse signals. *Biometrika*. 2010;97(2):465-480. doi:10.1093/biomet/asq017
